# The effect of weather conditions on scores at the United States Masters golf tournament

**DOI:** 10.1007/s00484-023-02549-6

**Published:** 2023-09-07

**Authors:** Harry Jowett, Ian D. Phillips

**Affiliations:** School of Geography, Earth and Environmental Sciences, University of Birmingham, Edgbaston, Birmingham, B15 2TT UK

## Abstract

This paper investigates the sensitivity of golfers’ performance to meteorological conditions at the men’s US Masters tournament over the 40-year period 1980–2019. The mean and standard deviation of round scores are related to local temperature, humidity, wind speed and direction, and concurrent and antecedent precipitation. Mean scores are more dependent on weather conditions than the variability of scores in a given round. The best predictor of mean scores is the wet-bulb temperature in rounds one and two, and the zonal wind speed in rounds three and four. Across both sets of rounds (1 and 2, and 3 and 4), the wet-bulb temperature is a better predictor of mean scores than the air temperature, which implies that atmospheric moisture content affects scores. In general, golfers take fewer shots and so perform better in warmer and calmer conditions. The synergestic effect of several weather variables explains over 44% of the variance in mean scores. Mean meteorological conditions during play are a much better predictor of the players’ average performance than the standard deviation of the weather variables. The golfers’ performance becomes more variable in cooler conditions with a wider range of scores. Precipitation during play and the dampness of the ground (as quantified by rainfall up to ten days before play) do not have a consistent and statistically significant effect on the competitors’ performance. In short, this paper demonstrates that golf scores are dependent on weather conditions.

## Introduction

As golf is an outdoor sport, enthusiasts pay careful attention to weather forecasts before major golf tournaments. In a round of golf, the ball spends most of its time on the ground. However, relative to other sports, a golf ball is airborne for longer periods of time and over greater distances. This means that the combination of ground and atmospheric conditions is likely to affect golf to a greater extent than in most other sports. Accordingly, certain conditions are likely to make scoring more difficult. Thornes (1977) describes golf as a ‘weather advantage’ sport. Each competitor starts and finishes at different times, which means that players can experience different meteorological conditions during their rounds on the same day. Consequently, players that begin their rounds on a fine clear morning have a clear advantage over those players that might have to deal with strong winds and heavy rain in the afternoon. Golf is also a ‘weather interference’ sport (Thornes 1977), where extremes of precipitation, visibility and wind speeds can halt play. This happened at the 1983 US Masters Tournament, where the second round was delayed by one day. This creates problems for some competitors because they then must play two rounds in a single day to get the tournament back on schedule. Some players can take up to five hours to play a round. The 1951 and 1953 Masters’ Champion Ben Hogan struggled to play more than 18 holes per day due to complications from injuries sustained in a car accident (Dodson 2013); and one could presume that if interfering weather had intervened in his winning years, he may not have found success.

**Fig. 1:**
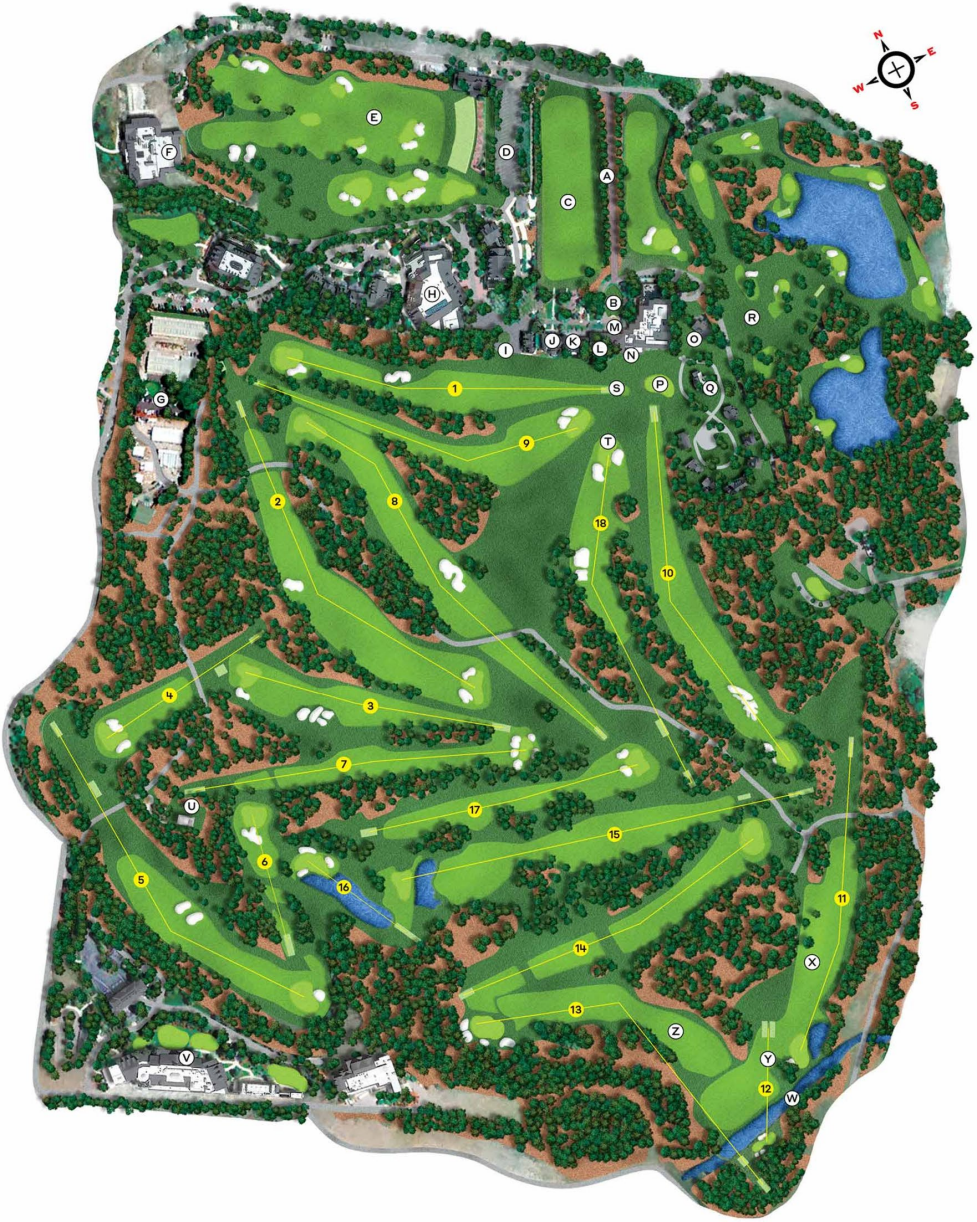
Plan of Augusta National Golf Course. The longest hole is number two (length = 526 m) and the shortest is number 12 (142 m). (Source: golf.com/travel/augusta-national-course-map-buildings-landmarks/)

*The Masters* takes place over 4 days in April. It is generally considered the hardest and most prestigious competition to win (Burke 2012). Moreover, it is the ideal tournament to study the effects of weather conditions on golf because it is held at the same course every year: the Augusta National Golf Club in Georgia, USA (Fig. 1). The fact that the tournament takes place around the same time every year is also advantageous because it means that golfers’ performance is not affected by seasonal variations in the area’s climate. The closest equivalent major tournament where competitions are played regularly at the same course is *The British Open*. However, this tournament is played at St. Andrews (Scotland) once every five years. Accordingly, *The Masters* tournament provides a richer data archive because 160 days of play are available over 40 years compared to only 40 days for *The British Open* at St Andrews.

The aim of this paper is to determine the relationship between meteorological variables and the average and variability of men’s *Masters* golf scores. This paper is the first attempt to examine quantitatively the association between golf scores and weather conditions. By doing this, it will determine whether the weather has a large or small effect on scores. This paper’s results will be of interest to gambling markets. Betting companies shorten the odds for low scoring markets, where forecasted conditions are similar to conditions that have favoured low scoring in the past. Moreover, this paper will determine whether competitive advantage exists in different weather conditions.

This paper is structured as follows. Firstly, previous relevant research is summarised before data and methods are described in the ‘Materials and methods’ section. Results are tabulated in the ‘Results’ section and discussed in the ‘Discussion’ section. Conclusions are drawn in the ‘Conclusion’ section.

### Research background

The wind has the largest effect on a player’s airborne shot (Malik and Saha 2020). The wind velocity affects the aerodynamic forces acting upon the ball by changing its relative air speed. This affects the ball’s magnitude of deceleration, which changes the carrying distance of the ball. The wind speed also affects the magnitude of variation, which stems from undue spinning of the ball. This means that an impure strike of the ball results in more error in the shot direction when a headwind is stronger and vice-versa for a tailwind. A strong crosswind will cause perturbations, either to the left or the right of the intended shot track, meaning professionals often aim into the origin of the wind direction to account for the perturbations. Otherwise, for all wind directions, players can hit shots with less backspin and at a shallower angle, which causes the ball to hang low in the air whilst still retaining the same carrying distance. Such ‘stinger’ or knock-down shots are designed to reduce the wind’s effects by taking advantage of lower wind speeds near the ground. However, mitigating for wind poses a greater risk for players because these types of shots are unorthodox (Malik and Saha 2020). This is due to the Magnus effect, whereby a spinning object moving through the air is deflected in a different manner to a non-spinning object. The golf ball’s dimpled pattern amplifies this effect. In addition, the turbulent flow behind a dimpled ball reduces the drag coefficient acting upon the ball. This allows a golf ball to fly more than twice the distance as a smooth ball of the same size (Bearman and Harvey 1976; Burglund and Street 2011).

Relative wind direction is also crucial in determining the aerodynamic coefficients of approach shots where accuracy is more important than distance. A headwind can cause the ball to slow much more quickly. This increases the landing angle, which makes accuracy and obstacle avoidance more difficult. By contrast, a tailwind decreases the landing angle. This means that the ball rolls farther on landing, which makes distance control on fast greens harder (Malik et al. 2018). The effects of head and tailwinds become greater when a player uses shorter clubs, where the intention is to hit the ball at a larger angle to the ground. Approach and pitch shots are up to twice as affected by the wind compared to driving shots, where the ball has a lower loft angle.

The orientation of the holes on a course is critical because a strong wind in one direction may hinder players whilst another direction might make scoring easier. Table 1 shows the orientation of each hole at *The Masters*. Slightly more holes are orientated from north to south or south to north (ten holes) than west to east or east to west (eight holes).

**Table 1:** The playing orientation of each hole at Augusta National Golf Club*.* The orientation is from the point of teeing off to the hole. The direction indicated is thus the tail wind

Hole	Playing orientation (degrees)	Wind direction	Tends to west–east (Zonal, Z) or north–south (Meridional, M)
1	125	EES	Z
2	330	NNW	M
3	125	SE	Z
4	85	E	Z
5	330	NNW	M
6	190	S	M
7	290	WWN	Z
8	160	SSE	M
9	305	NW	Z
10	10	N	M
11	40	NE	M
12	30	NNE	M
13	155	SSE	M
14	265	W	Z
15	105	EES	Z
16	160	SSE	M
17	265	W	Z
18	180	S	M

Whilst the wind’s effects on golf players are well recognised, other meteorological variables have received far less attention. It makes sense to postulate that air temperature affects scoring because it changes the air’s density and so the aerodynamic forces acting upon a ball.

Precipitation can increase air resistance for a ball and so reduce its range. More importantly, it alters the dampness of the ground. This increases the green’s softness, which can reduce the rolling momentum of the ball (Linde et al. 2011). A moist putting green makes the green play more slowly (Brede 1991) and could lead to higher scores for players that hit shorter tee shots. However, a moist green has one benefit in the sense that the ball will not roll as far off-line when compared to a dry green. Softer ground also tends to benefit players on shots approaching the green, as the ball will ‘plug’ in its landing position or move only slightly after impact. This can reward poorly struck approach shots (Drake 2014). Given that around 70% of shots are played in and around a putting green (Lodge et al. 1991), it is therefore surprising that rainfall’s influence on golf has been neglected to date. Most golf balls have a coating of a hydrophobic thermoplastic called polyurethane. In damp conditions, moisture introduces a lubricant between the ball and iron club. This means less spin and so less control for a professional golfer.

A golf player’s comfort is determined largely by the weather conditions. Anomalously cold conditions have a detrimental effect on a competitor’s ability to grip the club. Grip will also be influenced by wind (especially in cold weather) and precipitation. In cold weather, players wear additional layers of clothing, which decrease a competitor’s freedom of movement. It is widely recognised that the performance of athletes and cyclists (Galloway and Maughan 1997) is impaired in high temperatures and humidities. Less energetic sports such as golf might also be affected by extreme conditions. High humidity results in stuffy conditions, causing higher perspiration and heat stress. The hydration demands on a player are increased (Liljegren et al. 2008), as the body’s attempt to cool by sweating is prevented by the lack of evaporation. Humidity affects how far a golf ball will travel. Given the same temperature and pressure, humid air is lighter than dry air and so a golf ball will generally travel farther when the air is more humid.

In golf, a lower (higher) score equates to a better (worse) player. Variations in weather conditions at the hourly scale could affect competitors’ scores. This is more likely to be the case when conditions change during a player’s round, that is, on timescales of around two hours. If the weather changes on timescales of many hours (e.g., morning versus afternoon), then average scores across all players may not vary so much. This is because around half the field would play in more favourable conditions whilst the other half plays during less favourable conditions. Days with changeable conditions might have higher average scores, as players have constantly to adapt their approach. For example, changes in wind speed and direction may create the need for different styles of shot and playing style. Similarly, a day where the weather is settled should lead to stability in players’ technique, which may make competitors feel more comfortable and allow them to score lower.

## Materials and methods

This paper will mostly use correlation and regression techniques to quantify the effect of weather conditions on golf scores over the 40-year period from 1980 to 2019. The decision was taken not to analyse all *Masters*’ tournaments since 1934 because there are significant differences between pre-1980 and modern-day players. This reflects the effect of new club and ball technologies, rule and course changes, and physique changes amongst competitors. Moreover, during the period 1980–2019, metal drivers became the norm as opposed to the old ‘woods’; golf balls underwent major technological changes; and players became more ‘professional’ in terms of their lifestyle, a change that accelerated markedly after Tiger Woods’ dominance of the game in the 1990s. Restricting the data analysis to 1980 to 2019 means that the results will be more applicable to the modern game of golf. They are thus more useful for the near future, rather than providing an analysis of a time when the sport was hugely different from today. The ‘modern era’ of golf started around 1995 and it would have made sense to analyse only tournaments from the mid-1990s onwards. However, this would have resulted in fewer than 30 tournaments being analysed. Thus, the study period is a compromise between reflecting the modern game of golf; and having an adequate sample size to ensure that the correlation and regression coefficients can be generalised to the population.

Four rounds are played during each year’s *Masters* tournament. Golf round scores were mostly obtained from Matt Courchene of datagolf.com. This database details each round score for every participant from 1983 to 2019. The years 1980 to 1983 were manually derived by downloading the scorecards from *The Masters*’ web-site. Georgia’s climate is defined as ‘humid subtropical’ by the Köppen-Geiger classification; it has hot and humid summers, and short but mild winters (Peel et al. 2007). Augusta’s mean annual precipitation total is 1107 mm; April is one of the driest months with an average of 71 mm. Conditions are generally pleasant in April; the monthly mean and maximum temperatures are 17.1 °C and 24.8 °C respectively. Given the time of year, the players can sometimes experience much cooler and warmer conditions. During the study period (1980 to 2019), the coldest day in the first half of April was 8th April, 1982 (mean daily value of 6.7 °C). The warmest day was 9th April, 2011 when temperatures averaged 29.4°C.

Meteorological data were acquired from the National Oceanic and Atmospheric Administration’s (NOAA) database. There is a weather station only 4 km from the golf course at Augusta Daniel Field Airport. However, this site only has hourly data available from 2005, so data from Augusta Bush Field Airport 12 km SSE of the course were used instead. Furthermore, the altitude (44 m) of Augusta Bush Field Airport is closer to the golf course (50-95 m) than Augusta Daniel Field Airport (125 m). During the 4 days of each year’s tournament, hourly observations of the following meteorological variables were abstracted: air temperature (*T*); wet-bulb temperature (*T*_w_); relative humidity (RH); wind speed and direction; sea-level pressure (SLP) and precipitation (PREC). Hourly data changed from being recorded on the hour to seven minutes to the hour in 1997. Sub-hourly readings were made in more recent years, but these were discarded to ensure consistency with earlier years. Trace rainfall readings were treated as a dry day. As the ground’s wetness might affect golf scores, rainfall totals were also calculated for the 2-, 5- and 10-day periods before the tournament started (antecedent precipitation). The wet-bulb depression (*T* − *T*_w_) was calculated from the air and wet-bulb temperatures; a higher wet-bulb depression indicates a drier atmosphere. Wind direction is a circular variable and is unsuitable for linear statistics. As a result, the wind was decomposed into its zonal (*u*: west to east) and meridional components (*v*: south to north). A positive value of *u* (*v*) means that the wind is blowing from the west (south). As this paper focuses on the quantitative modelling of mean scores from continuous variables, nominal and ordinal variables obtained from the present weather field (e.g., whether precipitation was light, moderate or high; the occurrence of mist and fog) were not considered. However, a comparison was conducted between scores obtained in totally dry tournaments and those that saw some rainfall.

Daily means were calculated by averaging the hourly values during the times of play (08:00 to 20:00). Each year’s tournament is planned to last for 4 days. Tournaments that enter a fifth day are indicative of significant weather-related delays. For these 5-day tournaments, archive research was performed to remove days when no play occurred. For all tournaments, qualitative research was used to determine which rounds were played over different and/or multiple days. When delays in play caused the same round to be played over 2 days, an average of the hourly conditions across the 2 days was calculated. This was acceptable, given that this paper considers the *average* performance of the *whole* field of golfers. In rounds one and two, there are typically between 85 and 100 golfers. For the tournaments held between 1980 and 2012, the top 44 players—along with any players tied with the 44th player—progressed to the final two rounds. The rules were changed in 2013; and currently the top 50 players and any ties, and any players that finished the second round within ten shots of the leader now make the cut. If the analysis had focused instead on specific players and holes of the course, then more time-specific meteorological data would have been required.

The readings abstracted for the tournament and antecedent conditions were typically for the fortnight before the third Monday in April. In 1984, the tournament took place a week later than usual and in 1983 heavy rain resulted in play being extended to the third Monday. The NOAA’s *Daily Summaries* were used to check the validity of the daily averages. Press reports were also used to check anomalous conditions such as 2007 (*T* < 10 °C).

Means and standard deviations were calculated for each round of each year’s tournament. This enables both the average and the consistency of the players’ performance to be evaluated.

Long-term trends in sports performance must firstly be removed before the effect of weather conditions can be evaluated. For instance, Morris and Phillips (2009) found a statistically significant downward trend in the winning times of the men’s Oxford-Cambridge University Boat Race from 1949 to 2007. The changes previously discussed (e.g., changes in clubs and balls) might have caused the average round to be completed in fewer or more shots over the period from 1980 to 2019. For instance, the longest average driver in 1987 (279 yards) was Davis Love III, yet in 2019, this performance would not have even made it into the top 200. Linear regression showed that there were no long-term statistically significant (*p* < 0.05) trends in mean scores (for all rounds *R*^2^ = 0.007, *F* = 1.095, *p* = 0.297) and standard deviations (for all rounds *R*^2^ = 0.0004, *F* = 0.062, *p* = 0.804). There were also no significant trends in mean scores and standard deviations over time for rounds one and two collectively (mean scores: *R*^2^ = 0.004, *F* = 0.329, *p* = 0.568) and for rounds three and four combined (mean scores *R*^2^ = 0.015, *F* = 1.182, *p* = 0.280). Given the factors and changes previously discussed, the absence of statistically significant trends is surprising. However, the least-squares regression line for mean scores always has a downward slope, which indicates some improvement in golfers’ performance over time, all be it one that is not statistically significant.

The golfers’ performance differs between the four rounds of the tournament (Table 2). This effect must be removed before the golfers’ performance is related to the weather conditions. Golfers in rounds three and four take on average 1.24 fewer shots compared to the first two rounds. This is because the worst performing golfers are eliminated after the second round. The higher standard deviations (Table 2) in the first two rounds are indicative of more variable competitors. A Student’s *t* test reveals that there is a statistically significant difference in the means (*t* = 6.764, *p* < 0.001) and standard deviations (*t* = 7.418, *p* < 0.001) when the first two rounds are compared to rounds three and four. In the light of this, the effect of meteorological conditions was quantified for rounds one and two; and then separately for rounds three and four. There are only small and statistically insignificant differences in the round means and standard deviations between rounds one and two (*t* = 1.582, *p* = 0.118), and between three and four (*t* = 0.559, *p* = 0.578) (Table 2). These rounds therefore could be grouped together to create larger sample sizes, which may provide more meaningful results.

**Table 2:** The mean and standard deviation of the number of shots for the four rounds of the US Masters from 1980 to 2019*.* A proficient golfer is expected to take 72 strokes to complete one round of the Augusta course

Round	Mean	Standard deviation
1	74.23	3.49
2	73.83	3.36
3	72.86	2.99
4	72.71	3.03

Non-normally distributed variables (2- and 10-day antecedent rainfall, rainfall during a playing day and standard deviation of rainfall during a playing day) were transformed by taking the common logarithm. Unfortunately, this process only normalised the 10-day antecedent rainfall.

This paper’s aim is to predict the mean and standard deviation of the golf scores (the dependent or *y* variables) from the weather variables (the independent or *x* variables). As relationships between sports performance and the weather can be non-linear (e.g., athletic performance is impaired in cold and hot conditions), a scattergraph was plotted between each pair of *x* and *y* variables to determine whether linear statistics could model the relationship satisfactorily. Pearson (Spearman) correlation coefficients were calculated between pairs of normally (non-normally) distributed variables. Stepwise multiple linear regression was then used to identify the combination of meteorological variables that account for the most variance in golf scores.

## Results

### Associations between mean round scores and weather conditions

Firstly, the association between the mean round score and the arithmetic mean of each meteorological variable when play is taking place is considered (Table 3). A positive (negative) correlation indicates that the performance of the golfers deteriorates (improves) as that particular weather variable increases. In rounds one and two, scores tend to be lower when conditions are warmer with higher pressure and a stronger southerly wind. Golfers tend to perform worse when the zonal and vector wind speeds increase. The directionality of these relationships is preserved in rounds three and four when only the best performing golfers remain in contention. Whilst relationships with air and wet-bulb temperature are still statistically significant, the effect of these variables has weakened. It seems that the weakest players in the tournament are more affected by extremes of warm and cold. Relationships with concurrent and antecedent rainfall are weak, and are often inconsistent between rounds.

**Table 3:** Correlation coefficients between mean round scores and the arithmetic mean of each meteorological variable when play is taking place. One star indicates that the relationship is statistically significant at the 95% confidence level; two stars are the 99% level

	Rounds one and two	Rounds three and four
Pearson’s correlation		
*T*	− 0.56^**^	− 0.24^*^
*T*_w_	− 0.58^**^	− 0.35^**^
RH	− 0.21	− 0.36^**^
Wet bulb depression (*T* − *T*_w_)	0.16^*^	0.29^**^
Wind speed	0.44^**^	0.52^**^
Zonal wind speed (*u*)	0.36^**^	0.54^**^
Meridional wind speed (*v*)	− 0.41^**^	− 0.10
SLP	− 0.32^**^	− 0.08
Logarithm of 10-day antecedent PREC	− 0.05	− 0.22^*^
Spearman’s correlation		
PREC	0.03	− 0.15
Five-day antecedent rainfall	0	− 0.23^*^
Two-day antecedent rainfall	0.24^*^	− 0.14

Secondly, the relationship between the mean round score and the standard deviation of each meteorological variable when play is taking place is quantified (Table 4). A positive (negative) correlation shows that the golfers’ performance deteriorates (improves) when conditions are more (less) variable on that day. The only statistically significant (*p* < 0.05) associations are in rounds one and two, where competitors tend to perform better when the air temperature varies more during a day’s play. Conversely, they tend to perform worst when the meridional wind speed is more variable.

**Table 4:** Pearson’s correlation coefficients between mean round scores and the standard deviation of each meteorological variable when play is taking place. One star indicates that the relationship is statistically significant at the 95% confidence level; two stars are the 99% level

	Rounds one and two	Rounds three and four
*T*	− 0.30^**^	0.12
*T*_w_	− 0.19	0.11
RH	− 0.20	− 0.02
Wind speed	0	0.21
*U*	0.13	0.18
*V*	0.31^**^	0.21
SLP	0.11	0.08

### Associations between standard deviation of round scores and weather conditions

The weather might affect the consistency of the players’ performance in a given round. A higher standard deviation indicates that the golfers’ scores are more variable in that particular round. Correlation coefficients were calculated between the logarithm to the base ten of the standard deviation of the round scores (the original distribution was positively skewed), and the arithmetic mean and standard deviation of hourly values of each meteorological variable when play is taking place (Table 5). A positive (negative) correlation indicates that the competitors’ scores become more (less) variable as that particular weather variable increases. The weather has less effect on the standard deviation of the scores than the mean score. Arithmetic mean air temperature and arithmetic mean relative humidity are the only variables with statistically significant (*p* < 0.05) relationships across both the first and last two rounds. A higher air temperature tends to reduce the spread of the golfers’ scores. The effect of mean relative humidity is contradictory (positive in rounds one and two, and negative in rounds three and four). More variable rainfall and pressure conditions during the first two rounds tend to result in a greater dispersion in the golfers’ scores.

**Table 5:** Correlation coefficients between the logarithm to the base ten of the standard deviation of round scores, and the arithmetic mean and standard deviation of each meteorological variable when play is taking place. One star indicates that the relationship is statistically significant at the 95% confidence level; two stars are the 99% level

	Rounds one and two	Rounds three and four
Pearson correlation		
Arithmetic mean of weather variable		
*T*	− 0.22^*^	− 0.26^*^
*T*_w_	− 0.03	− 0.33^**^
RH	0.29^*^	− 0.25^*^
Wet bulb depression (*T* − *T*_w_)	− 0.31^**^	0.20
Wind speed	− 0.09	0.01
*U*	− 0.18	0.15
*V*	0.08	− 0.20
SLP	0.03	− 0.09
Logarithm of 10-day antecedent PREC	− 0.11	− 0.06
Standard deviation of weather variable		
*T*	− 0.20	0.12
*T*_w_	− 0.11	− 0.01
RH	− 0.15	0.18
Wind speed	− 0.17	0.09
*U*	− 0.13	0
*V*	− 0.10	0
SLP	0.34^*^	− 0.05
Spearman’s correlation		
PREC	0.27^*^	− 0.03
Five-day antecedent rainfall	− 0.04	0.09
Two-day antecedent rainfall	0.06	− 0.01

### Modelling the association between mean round scores and weather conditions

**Fig. 2:**
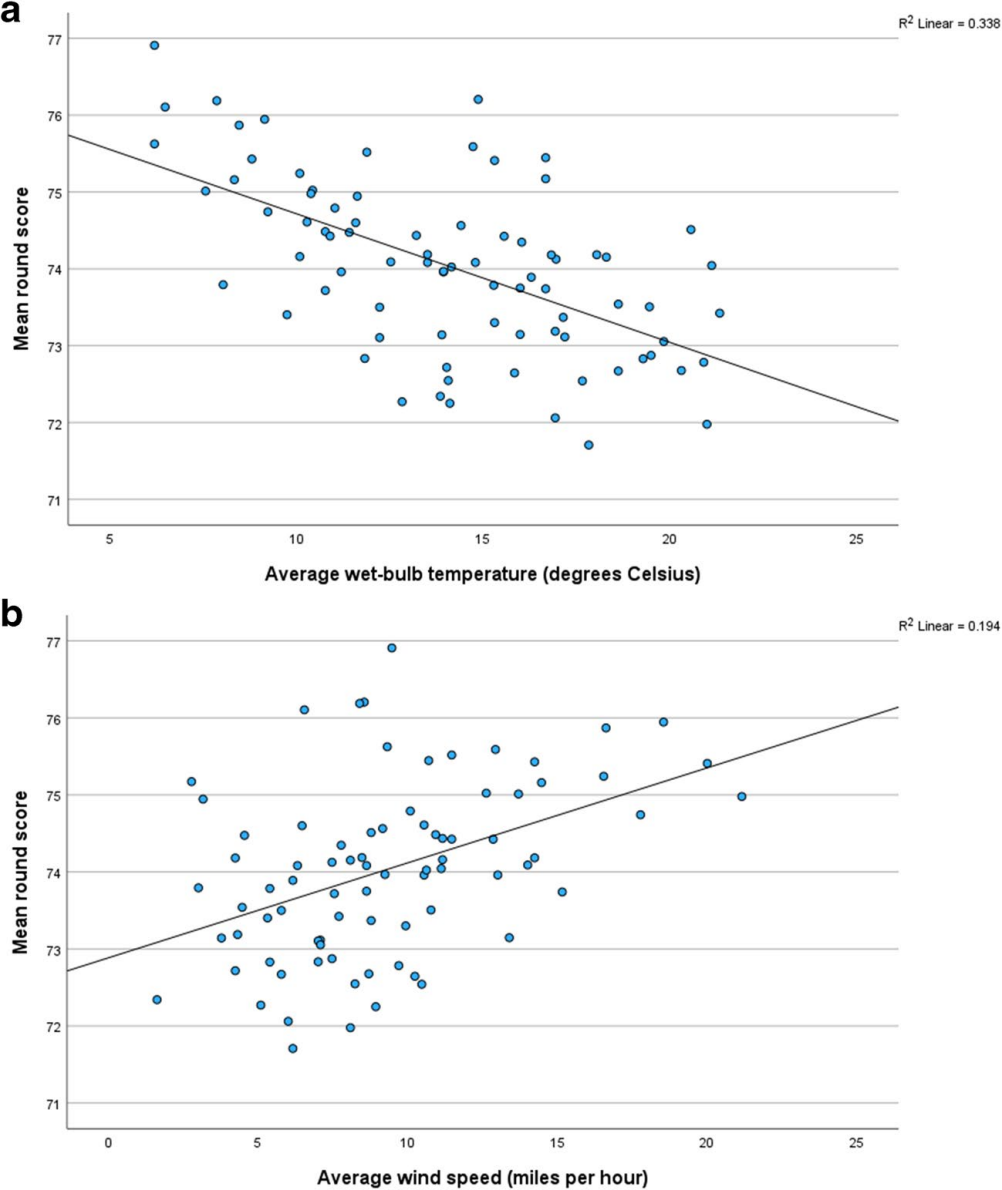
The association between the mean scores in rounds one and two and **a** average wet-bulb temperature during play and **b** average wind speed during play

**Fig. 3:**
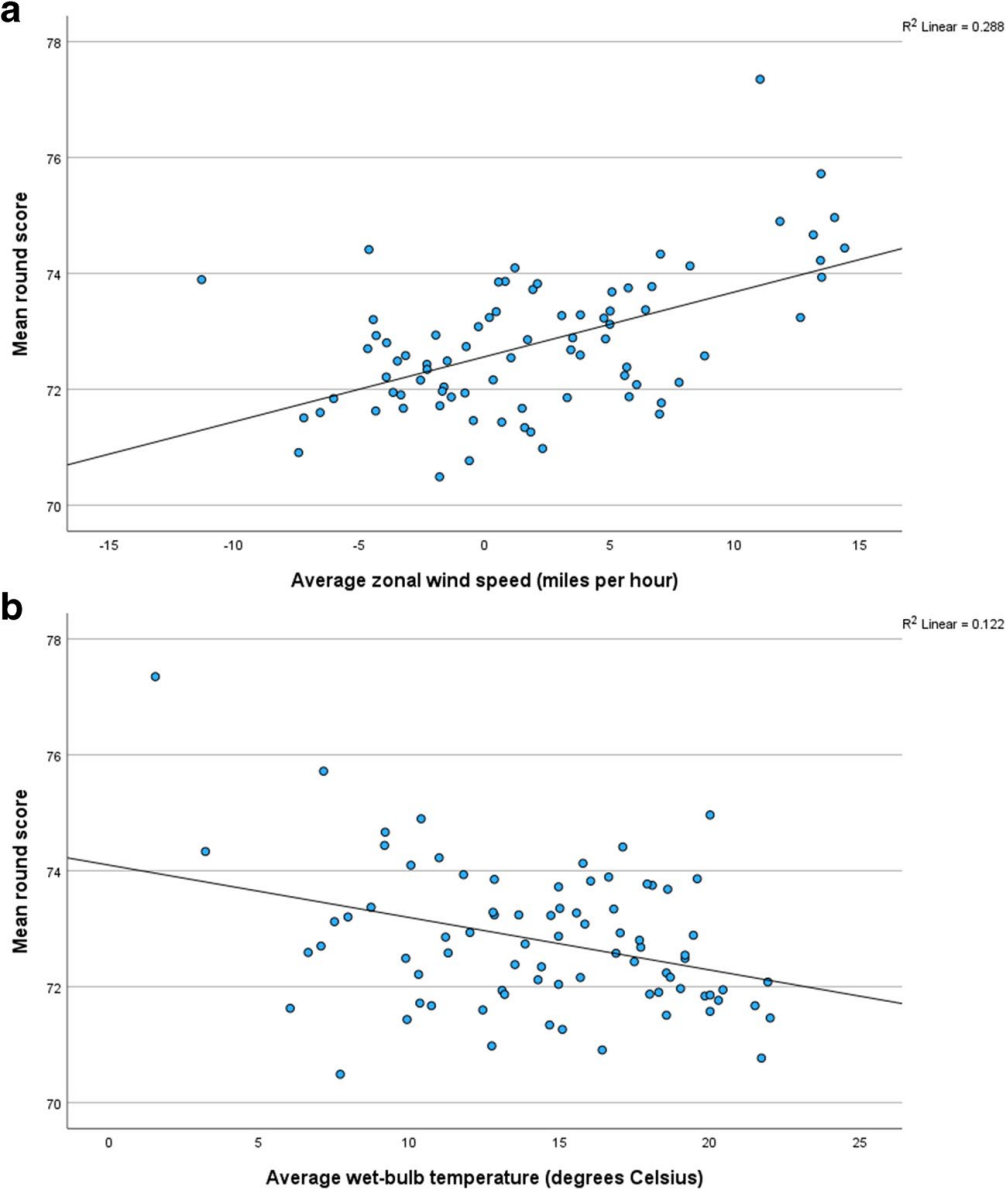
The association between the mean scores in rounds three and four and **a** average zonal wind speed during play and **b** average wet-bulb temperature during play

In light of the statistically significant relationships already discussed (Tables 3 and 4), it is possible to model the mean golf scores from the weather conditions. Scattergraphs of the most promising predictors (Figs. 2 and 3) revealed that the association between the meteorological variable and the score was linear.

A stepwise linear regression model was formulated to predict the mean scores in rounds one and two. This model contains three variables: average wet-bulb temperature, average wind speed and average precipitation during play. The model as a whole is statistically significant at the 0.001 level (*F* = 23.637, *p* < 0.001) and accounts for 46.2% (Adj*R*^2^ = 0.462) of the variance in golf scores. The model’s four coefficients were significant at the 0.01 level:

1 $$\mathrm{Mean}\;\mathrm{golf}\;\mathrm{score}=75.249-\left(0.150\times\mathrm{average}T_w\right)+\left(0.089\times\mathrm{average}\;\mathrm{wind}\;\mathrm{speed}\right)+\left(0.606\times\mathrm{PREC}\right)$$

Units: temperature (°C), wind speed (miles per hour) and precipitation (mm).

In rounds one and two, the average wet-bulb temperature is the best predictor of mean scores. It accounts for 33.8% of the variance (Fig. 2a), with scores decreasing in warmer conditions. Adding the average wind speed into the model increases the explained variance to 41.6% (Adj*R*^2^ = 0.416). Windier conditions tend to favour higher scores (Fig. 2b). Precipitation during play is the final step of the model. The variable’s positive regression coefficient indicates that the performance of golfers tends to deteriorate in wetter conditions. The inclusion of precipitation in the model is surprising because this variable does not have a statistically significant association with mean round scores (*r*_s_ = 0.03; Pearson’s *r* = 0.175, *p* = 0.120). A variable that is insignificant on its own can become significant when it is combined with other variables in a multiple regression model. This has happened here, which means that precipitation only has a significant effect on scores when it is considered in combination with the wet-bulb temperature and the wind speed. Scores are also significantly associated with the air temperature, and the *u* and *v* wind components (Table 3). However, these variables are omitted from the multiple regression model because their variability has already been explained by *T*_w_ or wind speed. They are thus redundant predictors.

The assumptions of the model were tested. A histogram and a Q-Q plot were drawn to check that the model’s residuals are normally distributed and centred around zero. This proved to be the case. The predictor variables of the model must be independent of one another. The estimates of the regression coefficients may be unreliable when predictors are highly correlated. The resulting extreme multicollinearity also makes it difficult to separate out the effects of the different predictors on the dependent variable. Menard (1995) advises that variables with tolerance values of less than 0.2 have potential multicollinearity problems. The three predictors in the model (*T*_w_, wind speed, PREC) all have tolerance values comfortably above this threshold (0.932 to 0.993); they are also close to the maximum tolerance of one (a predictor contains unique information). If the model is satisfactory, then it should predict low, medium and high golf scores with the same level of accuracy. This is the homoscedastic errors assumption. It is confirmed graphically by a random scatter of points when the standardised residuals are plotted against the standardised predicted values. The model’s errors are homoscedastic. There are two data points per year because round one and round two were entered into the model separately in order to increase the sample size. The Durbin-Watson statistic is 1.194, which means that the residual time series displays weak positive autocorrelation. The autocorrelogram of the residuals is statistically significant at lags one and two, although the level of autocorrelation is not extreme (< 0.4). In half of cases, some autocorrelation at lag one is inevitable because the same players in the same year are being compared. Three out of the 80 standardised residuals (3.75%) have an absolute value greater than two (> ± 2), which is broadly consistent with the 4.55% expected for a perfect normal distribution. Round two in 1983 and round one in 2005 are high leverage points because they experienced much wetter conditions than all the other tournaments.

For rounds three and four, stepwise regression selected four predictors: average zonal wind speed, average wet-bulb temperature, 5-day antecedent precipitation and average wind speed. This model accounted for 44.8% of the variance in golf scores (Adj*R*^2^ = 0.448, *F* = 17.06, *p* < 0.001, Durbin-Watson statistic = 1.597). The model’s five regression coefficients were statistically significant at the 0.05 level or better:

2 $$\mathrm{Mean}\;\mathrm{golf}\;\mathrm{score}=73.544+\left(0.072\times\mathrm{average}\;u\right)-\left(0.087\times\mathrm{average}\;T_{\mathrm w}\right)-\left(0.288\times\log_{10}5-\mathrm{day}\;\mathrm{antecedent}\;\mathrm{precipitation}\right)+\left(0.071\times\mathrm{average}\;\mathrm{wind}\;\mathrm{speed}\right)$$

All five predictor variables have statistically significant Pearson correlation coefficients with mean scores (Table 3). For each variable, the sign of the regression coefficient is consistent with its correlation coefficient. In rounds three and four, the zonal wind speed is the best predictor of scores and explains 28.8% of the variance. The golfers’ performance tends to deteriorate when there is a stronger wind blowing from the west (Fig. 3a). Adding the wet-bulb temperature into the model increases the variance explained to 33.3% (Adj*R*^2^ = 0.333). Like the first two rounds, competitors tend to take more (fewer) shots in cooler (warmer) weather (Fig. 3b). The model’s residuals are normally distributed and homoscedastic. The tolerance values of the predictor variables vary between 0.610 and 0.871, which satisfies the independence assumption. Three out of the 80 standardised residuals (3.75%) have an absolute value greater than two (> ± 2), which is broadly consistent with the 4.55% expected for a perfect normal distribution. With the exception of lag nine (five years apart), the residual time series is random. There was one high leverage point: round three in 1985. This leverage point is explained by the high wind speeds during play.

### Modelling the association between the variability in round scores and weather conditions

The variability in round scores (as measured by the logarithm to the base ten of the standard deviation) is significantly associated with several meteorological variables (Table 5). Two sets of stepwise linear regression models were developed for the first and last two rounds to predict the standard deviation (logged) of the players’ scores. The first set used the mean values of the weather variables and the second set used the standard deviation of the hourly values of the meteorological variables when play was in progress.

For rounds one and two, the model containing the mean values contained only one variable: the wet-bulb depression (Table 6). There is a negative relationship, which means that the golfers’ performance becomes more variable when the atmosphere is more humid (lower wet-bulb depression). For rounds three and four, the stepwise model contained the wet-bulb temperature only. The players’ performance tends to become more variable when the wet-bulb temperature decreases.

**Table 6:** Predicting the standard deviation of the players’ round scores from the arithmetic means and standard deviations of the weather variables

Round number	Equation	^*^R^2^ or Adj*R*^2^	*F*	*p*
From mean values				
1 and 2	log_10_ standard deviation = 0.568 − (0.006 × average wet-bulb depression °C)	0.091	7.842	0.006
3 and 4	log_10_ standard deviation = 0.527 − (0.004 × average wet-bulb temperature °C)	0.111	9.762	0.003
From standard deviations				
1 and 2^**^	log_10_ standard deviation = 0.508 + (0.678 × standard deviation of hourly precipitation mm) + (0.011 × standard deviation of hourly sea-level pressure mb)	0.247	13.935	< 0.001
3 and 4	-	-	-	-

^*^For a simple model with only one predictor variable, *R*^2^ is quoted. For a model containing multiple predictors, Adj*R*^2^ is given

^**^The tolerance value of the predictor variables is 0.934, which means that multicollinearity is not a problem in this model

In the second set of models, the standard deviations of the weather variables are the predictor variables. For rounds one and two, the model contains two variables: hourly precipitation and hourly sea-level pressure. The golfers’ performance becomes more variable when a tournament is played in conditions that fluctuate between dry and wet (Table 6). The residuals of this model are approximately normally distributed. A Durbin-Watson statistic of 1.393 indicates that there is some positive correlation between successive residuals, but an autocorrelogram reveals that the level of autocorrelation is not severe (< 0.4). It was not possible to derive a statistically significant model for rounds three and four.

### The cumulative effect of weather-related disruption

**Fig. 4:**
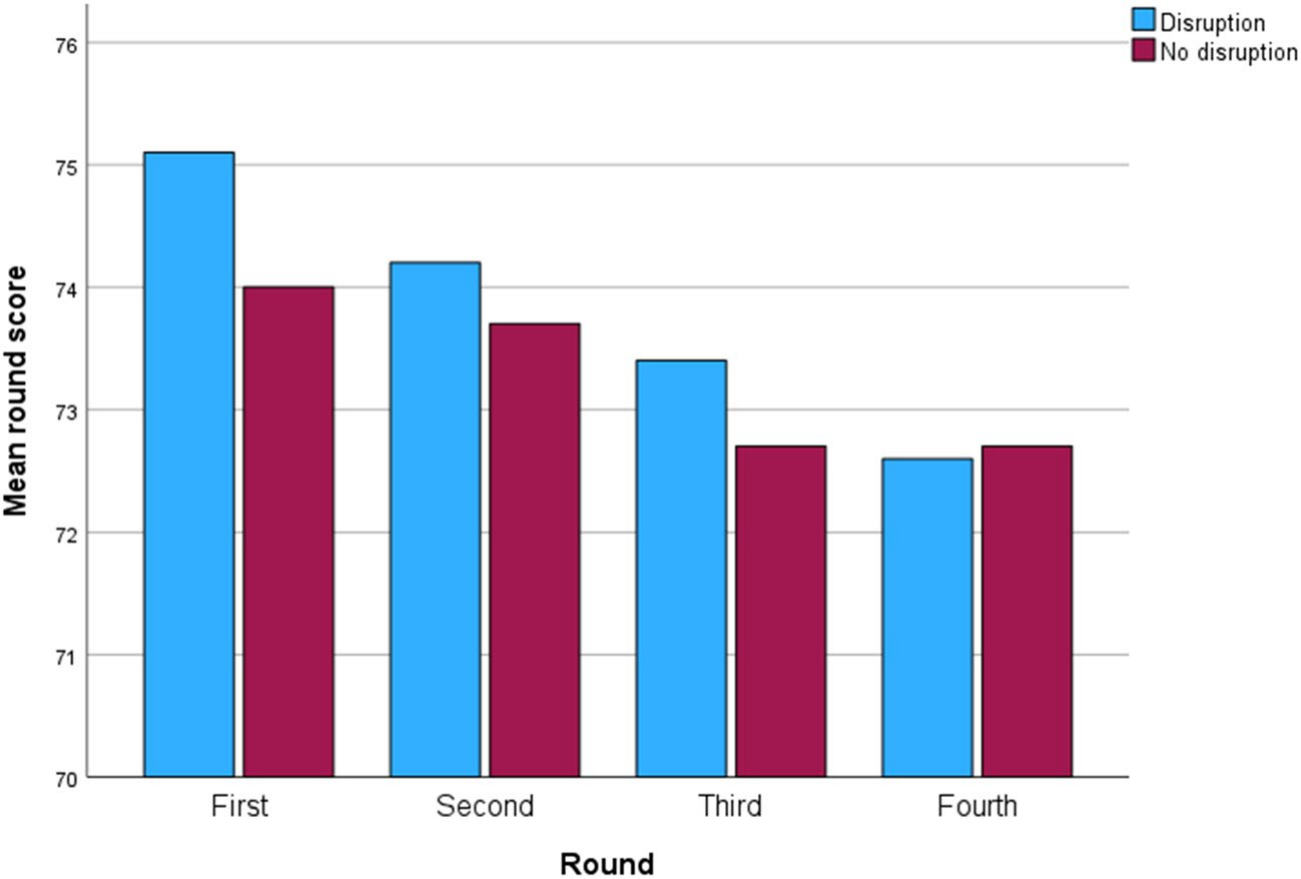
The mean round scores for tournaments with and without weather-related disruption. Note that the *y* axis starts at 70 and not 0. The number of years with disruption was as follows: round one (8 years), round two (9), round three (9) and round four (5)

For each round, a comparison was performed between those tournaments that were disrupted by delays and those that saw no disruption. Disruption occurs in three main ways: a round starts late because the previous round has not finished; play is suspended due to rain, thunderstorms or inclement weather; or play is suspended for the day owing to poor light. Weather-related disruption has the greatest effect in the first round, with golfers taking 1.1 more shots on average in years when play is disrupted by the weather (Fig. 4). In rounds two and three, competitors take 0.5 and 0.7 more shots respectively. However, the difference is only statistically significant at the 0.05 level in round one (*t* = 2.307, *p* = 0.027). Apart from round three, disruption also slightly increases the dispersion (as measured by the standard deviation) of the players’ scores. For rounds one and two, the increase in the variability of scores is significant at the 90% confidence level.

## Discussion

As expected, several meteorological variables had significant correlations with mean golf scores. The best predictor in rounds one and two (three and four) is average wet-bulb temperature (average zonal wind speed). Whilst the best predictor differs, wet-bulb temperature and zonal wind speed are statistically significant at the 99% confidence level in both sets of rounds.

Golf scores have a statistically significant (*p* < 0.05) negative association with air and wet-bulb temperatures over both sets of rounds. This means that players tend to take fewer shots in warmer conditions when the air density is lower. The lower air density allows the ball to fly further as the resistance upon it is lower (Baek and Kim 2013). Warmer temperatures may also help relax a player’s muscles, further improving their range of motion. Another explanation might be that players may choose to wear less clothing when the temperature is warmer, making their range of movement freer than if they are impeded with layers of warm clothing (Thornes 1977). However, at elite level tournaments, competitors are provided with professional sports clothing, which is designed not to hinder movement. The performance of golfers is more sensitive to variations in air and wet-bulb temperatures in the first two rounds than in the last two rounds. This probably reflects the wider ability range of the players during rounds one and two with the worst competitors being eliminated at the end of the second round. The greater preponderance of lower skilled, out-of-form or older players in these earlier rounds whose performance is more sensitive to temperature changes might explain the stronger relationship. The correlation coefficients for rounds one and two were calculated using all players, that is, those eliminated at the end of the second round and those that made the cut and so progressed to the final two rounds of the tournament. For rounds one and two, it would be interesting to calculate a correlation coefficient for each group of competitors. This would then enable the effect of weather conditions on the weaker players to be quantified more accurately because the real association is partly concealed when all players are considered.

The average wind speed is a good predictor of the players’ performance. This variable accounts for 19% (27%) of the variance in mean scores during the first (last) two rounds of play. The positive association between the two variables means that the performance of golfers tends to deteriorate in windier conditions. This finding is logical: stronger winds will change how the ball behaves in the air, adding additional challenges for competitors. The highest third round scores occurred in 2007 (mean round score = 77.4) and 2016 (mean = 75.7). In terms of mean speeds, these years were the third and second windiest respectively over the 40-year period. Both years saw gusts of up to 40 mph. Similarly, the worst performing fourth round (1999: mean round score = 75.0) saw the windiest conditions (mean speed = 14.8 mph) over the period from 1980 to 2019. Conversely, the best performing fourth rounds (2018: 70.5 shots and 2015: 70.9 shots) took place in much calmer conditions with mean wind speeds of 7.8 mph and 5.3 mph respectively.

The zonal (*u*) and meridional (*v*) components allow the effects of wind direction to be quantified. Seven (nine) of the holes on the course (Table 1) are orientated from west to east (east to west). The fact that mean scores are positively related to the zonal wind speed (a stronger wind from the west tends to result in higher scores) is logical because a westerly wind is a headwind at more holes than it is a tailwind. The zonal wind speed (*u*) is also strongly correlated (*r* = 0.68, *p* < 0.001) to the vector wind speed. When the course experiences a westerly wind, it tends to be stronger, thus impairing performance. In addition, slightly more holes are orientated closer to N-S (10 holes) than W-E (eight holes) (Table 1). This means that across the entire course a strong zonal wind is more likely to be a crosswind than it is a head or tail wind. Whilst mean scores are inversely related to the meridional wind speed in both sets of rounds, the association is only statistically significant in rounds one and two. The negative association indicates that golfers tend to take fewer shots when the wind is blowing from the south rather than from the north. Eight holes have a southerly tailwind and eight holes have a northerly tailwind (Table 1), which suggests that the orientation of the holes is not the explanation. The inverse association is probably explained by the fact that southerly winds bring warmer conditions (Pearson’s *r* between *v* and air temperature = 0.505, *p* < 0.001), which have already been shown to improve golfers’ performance.

The players’ performance tends to deteriorate when atmospheric pressure is lower. This is most likely because lower pressure is associated with stronger wind speeds (*r* =  − 0.42, *p* < 0.001, *n* = 160), which were earlier shown to decrease golfers’ performance. This was confirmed by calculating a partial correlation coefficient: the statistically significant association between mean scores and SLP in rounds one and two (*r* =  − 0.32, *p* < 0.01) disappears when one controls for the effect of wind speed (*r* =  − 0.11, *p* = 0.32). Furthermore, lower pressures could also indicate an approaching depression with its associated unsettled weather conditions.

The effect of the variability of meteorological conditions during play (as quantified by each weather variable’s standard deviation) on round scores was investigated. It was hypothesised that scores would be negatively related to the standard deviation. This is because a lower standard deviation indicates more consistent playing conditions, which means that players could play in a more similar manner throughout the day. The analyses disproved this hypothesis, as none of the variables’ standard deviations had a statistically significant relationship with scores across both sets of rounds. Mean conditions are a much better predictor of golfers’ performance than the variability in these conditions.

The golfers were expected to take more shots in wetter conditions. However, no statistically significant correlation was found between precipitation and scores in both sets of rounds. Student’s *t* tests between tournaments played in dry (rainfall = 0 mm) and non-dry (rainfall > 0 mm) conditions confirmed the absence of a statistically significant effect (*t* =  − 0.407, *p* = 0.685 for rounds one and two; *t* = 1.419, *p* = 0.160 for rounds three and four). The fact that mean round scores show significant dispersion in dry conditions further suggests that precipitation is not the most important factor. Over 75% of the rounds are played in dry conditions, which means that the number of rounds played in very wet conditions is comparatively low. There have only been three tournaments (1983, 1989 and 2005) where the 4-day (5-day in 1983) rainfall total has exceeded 25 mm. The absence of a significant relationship might also be explained by the fact that play is sometimes suspended when moderate to heavy precipitation occurs (Spencer 2020). This is because of the moisture, bad light and risk of lightning strikes. Consequently, play is often halted when any sizeable precipitation falls, which means that its effects on the golfers’ performance cannot be investigated. For example, rain resulted in the first day’s play in 1982 being suspended at 4.23 pm. Thunderstorms saw the third round in 1992 and 2006 being halted for several hours. The dampness of the ground (as quantified by the amount of precipitation in the ten days before the tournament) did not have a consistent and significant effect on the golfers’ performance across both sets of rounds.

Mean golf scores reveal nothing about the consistency of the players’ performance in a given round. Hence, the standard deviation of the players’ scores was calculated. A higher standard deviation indicates less consistency and thus a wider range of ability. The air temperature was the only variable to have a significant and consistent effect (in this case a negative effect) on the standard deviation of the scores in both sets of rounds. This means that the range of players’ scores is greater in cooler conditions, which suggests that weaker players struggled more in colder temperatures.

Nearly half of the variance in mean round scores can be explained by variations in meteorological conditions (Adj*R*^2^ = 0.462 for rounds one and two; Adj*R*^2^ = 0.448 for rounds three and four). The multiple regression models for the two sets of rounds both contain the wet-bulb temperature and either the vector or zonal wind speed. Golfers tend to take fewer shots to complete the course in warmer and less windy conditions. It is surprising that the models do not contain the air temperature. However, across both sets of rounds, the wet-bulb temperature is a better predictor of mean scores than the air temperature (Table 3), which implies that atmospheric moisture content affects scores. Air temperature is not included in the models because it is a redundant predictor whose variance is largely already explained by the wet-bulb temperature (*r* = 0.81, *p* < 0.001 for rounds one and two; *r* = 0.89, *p* < 0.001 for rounds three and four). The negative effect of increasing wind speed agrees with the findings of Malik and Saha (2020). Whilst the zonal wind speed is the best predictor of golf scores in rounds three and four, the best predictor of mean scores in the first two rounds is the wet-bulb temperature. This reflects the increased sensitivity of the weaker players to cooler conditions in the earlier rounds. The multiple regression model for rounds one and two also contained precipitation during play, with the positive regression coefficient suggesting that competitor performance deteriorates in wetter conditions. However, golf scores did not have a statistically significant association with concurrent precipitation (*r* = 0.03; see Table 3), which suggests that this variable only becomes significant when it is combined with the other predictor variables in the model. Five-day antecedent rainfall appears in the multiple regression model for mean scores in rounds three and four. The variable’s negative regression coefficient implies that golfers perform better when the ground is softer. This could reflect the fact that softer ground tends to benefit players on shots approaching the green because the ball will ‘plug’ more into its landing position. Softer ground also results in slower green speeds, which usually makes putting easier given the severe slopes of the greens at Augusta National Golf Club. This could be another reason why round scores are not significantly related to concurrent rainfall.

The standard deviation of the players’ scores is less sensitive to weather conditions than the mean score (Table 6). Even the best regression model (rounds one and two) explained less than one quarter of the variance in the competitors’ standard deviation (Adj*R*^2^ = 0.247). In rounds one and two, the golfers’ performance became more variable as the standard deviation of precipitation increases. This supports the notion of ‘weather advantage’ and ‘weather interference’ (Thornes 1977) because greater temporal variability in precipitation means that some competitors experienced rain in their rounds whilst others did not. This would then increase the variability in the players’ scores.

Future work could examine the effect of weather on golfers of different abilities. Those invited to compete in the *Masters* are all highly skilled players. A competitor’s performance is likely to relate to their form at the time, their familiarity with the course, and their overall health and well-being. Analyses could be performed using different age groups (e.g., the older golfers would be mostly past champions). The performance of golfers that qualified through amateur competitions could also be studied. Hourly meteorological data are available at Augusta Bush Field Airport from 1948 onwards. A companion study could be conducted comparing the performance of players over the 32 years from 1948 to 1979 with the current paper’s results for the modern era of golf. The same methodology could also be applied to The Players’ Championship, which is considered the fifth major of the season. The advantage of studying the Players’ Championship over the other majors is that it has been played annually at the same course (Ponte Vedra Beach in Florida) since 1974.

This paper has investigated the effect of concurrent and antecedent precipitation totals on golfers’ scores. Future research could use rainfall totals in conjunction with the present weather field to identify periods of rain and drizzle. The present weather field also has a code for whether the precipitation was light, moderate or heavy; and would allow the effects of mist and fog on competitors’ performance to be quantified.

This paper has quantified the effect of weather conditions on players’ mean scores during a complete round of golf (18 holes). A proficient golfer is expected to take 72 strokes to complete one round of the Augusta course. The par (the number of strokes that a proficient golfer should take) of the holes varies between three and five. It is possible to obtain a given score in different ways. For example, a score of 72 could be achieved by making 18 pars; or by making six birdies (one stroke under par), six bogeys (one stroke over par) and six pars. Future work could perform a hole-by-hole analysis to determine whether certain weather conditions result in significantly more birdies, bogeys, eagles (two under par), albatrosses (three under par) at each hole. This is now possible with the advent of technologies such as ShotLink, which collects and disseminates “scoring and statistical data on every shot by every player in real-time” (shotlink.com). Studying specific holes would also provide a more detailed insight into the effects of wind and dampness of the ground on scoring. The additional qualitative information provided by the present weather field would prove invaluable in this regard.

## Conclusion

This study has shown that scores at the US *Masters* golf tournament are influenced by meteorological conditions, namely, the air temperature, wind speed, wind direction and the saturation of the air. Correlation analysis revealed several meteorological variables whose mean values during play were moderately related to mean round scores. Regression models were then used to quantify the combined effects of several weather variables on the mean and standard deviation of round scores. In general, golfers’ performance improves in warmer and less windy conditions. Weather conditions collectively influence mean scores to a greater degree than the standard deviation of players’ scores in an individual round.

Competitors at *The Masters* may be interested in the fact that certain meteorological conditions make lower scores more likely. For example, if a player knew that temperatures were to increase throughout the day, then they may look to play more aggressively in order to spend their next day's round in the best possible conditions for scoring. Bookmakers currently use models to give odds of the percentage chance of certain occurrences on the Augusta course (e.g., the number of bogeys, birdies, holes-in-one; the ‘cut’ score) during a day’s play. They may want to incorporate the predictor variables from the regression models in this paper to refine their models.

This paper does not consider other impacts on scoring such as hole reconfigurations (the hole’s position on the green changes every year in order to adjust the level of difficulty) and the design of the whole course. The Augusta National Golf Club’s course has gained a total of 623 m over the past 40 years (Kelly 2019), which may have caused changes to the exposure of parts of the course. This paper also assumes that the conditions on the course are identical to those experienced at the Augusta Field Airport meteorological station 12 km away. Whilst this is a short distance on the synoptic scale, it is possible for the airport to be dry and the golf course to be wet and vice-versa. Given that the course covers 140 hectares, it is possible that it might be raining on some fairways and not on others.

The results presented in this paper are specific to the men’s US *Masters* over a period of 40 years. Generalisations about the weather’s effect on golf can only be made from an ensemble of similar studies of other tournaments held in different geographical settings. Moreover, there are other groups of professional players (e.g., female professionals, challengers, seniors) that may be affected differently by changing weather conditions at the same course. In addition, golf is enjoyed by millions of amateurs worldwide, who play the game completely differently to professionals. Questionnaires and interviews with golfers might also provide some valuable insights into the effects of weather of play.

In conclusion, this paper has demonstrated that the weather has a statistically significant effect on mean golf scores at the *Masters*. Nearly 50% of the variance in mean golf scores over 40 years of tournaments can be attributed to changes in weather conditions.

## Data Availability

The datasets generated during and/or analysed during the current study are available from the corresponding author on reasonable request.
